# Three in four smokers want to quit tobacco (reference to reassessing the smoking target in Japan): findings from the JASTIS2021 study

**DOI:** 10.1265/ehpm.23-00285

**Published:** 2024-05-14

**Authors:** Masayuki Sugihara, Takahiro Tabuchi

**Affiliations:** 1 Faculty of Medicine, Osaka University, 1-1 Yamadaoka, Suita-shi, Osaka 565-0871, Japan; 2 Cancer Control Center, Osaka International Cancer Institute, 1-69 Ohtemae 3-chome, Chuo-ku, Osaka 541-8567, Japan; 3 Division of Epidemiology, School of Public Health, Tohoku University Graduate School of Medicine, 2-1, Seiryo-machi, Aoba-ku, Sendai, Miyagi 980-8575, Japan

**Keywords:** Tobacco, Heated tobacco product, HTP, Smoking rate target, JASTIS, Japan, Smoking, Nicotine, Smoking prevalence

## Abstract

**Background:**

To enhance tobacco control in Japan, the government set a future smoking rate target (smoking prevalence = 12% by 2022) arguing that the “smoking rate target is expected if only smokers who want to quit smoking now, actually quit”. However, ideally both those wanting to quit now and those who wanted to in the past will succeed in the future. We aimed to re-define smokers who intend to quit and estimate their number. We also examined determinants of intention to quit, including daily tobacco consumption and tobacco use categories (exclusive combustible cigarette users, exclusive heated tobacco product (HTP) users, and dual (combustible cigarette and HTP)) users.

**Methods:**

Using data from the 2021 Japan ‘Society and New Tobacco’ Internet Survey, we analyzed 5,072 current smokers (had used combustible cigarettes or HTPs in the past 30 days) aged 20–80 years. Smokers who intend to quit were defined as total smokers who want to quit now, have previously attempted or previously wanted to quit. Log-Poisson regression models were used to calculate adjusted odds ratios (aORs) with 95% confidence intervals (95%CI) for intention to quit (current or current/past), adjusting for potential covariates such as tobacco use categories.

**Results:**

Among current smokers, 40.6% want to quit now, 27.0% have previously attempted and 9.0% have previously wanted to quit. Smokers of over 20 tobacco sticks/day are less likely to want to quit now than 1–10 /day (aOR = 0.79, 95%CI = 0.71–0.87) and less likely to intend to quit (both current and past) (aOR = 0.93, 95%CI = 0.88–0.98). Exclusive HTP and dual users were more likely to intend to quit (both current and past) than exclusive combustible cigarette users (aOR = 1.09, 95%CI = 1.04–1.14) and (aOR = 1.07, 95%CI = 1.03–1.12).

**Conclusions:**

In total, 76.6% of current smokers, were defined as having intention to quit (both current and past). Applying this percentage to the target calculation, Japan’s smoking rate target would be 3.9%, considerably lower than the current target of 12%. The Japanese government may have to revise the smoking rate target. Additionally, we found that the usage of HTPs reduces intention to quit smoking. These insights have implications for setting of smoking rate targets and regulating HTPs in different countries.

## Graphical Abstract

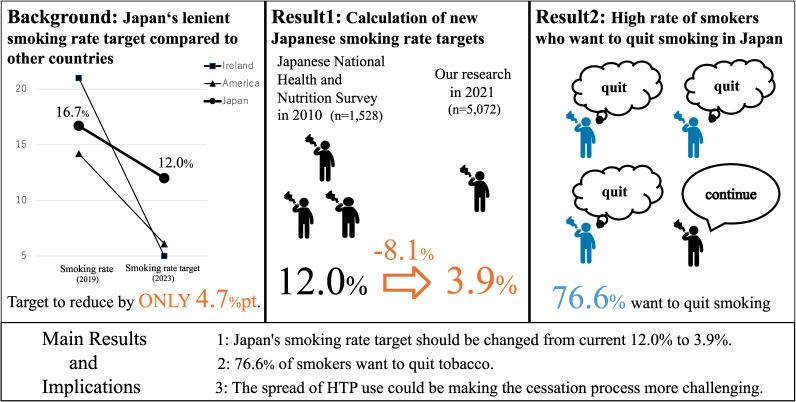

## Introduction

Smoking continues to be a significant global health issue, with tobacco causing 7.69 million deaths in 2019 (13.6% of all deaths) worldwide and 199,000 (14.2% of all deaths) in Japan [1]. Governments have implemented tobacco control policies aimed at reducing smoking rates by establishing a “smoking rate target”. In the US ‘Healthy People 2030’ strategy the smoking rate target has been set at: (smoking prevalence = 6.1% by 2030) [2]. The target in Japan is twice that at (smoking prevalence = 12% by 2022), although there was only a small difference in the current smoking rates between the two countries in 2019 (smoking prevalence = 14.2% in America [2] and 16.7% in Japan [3]). This suggests a possible flaw in Japan’s method for setting smoking rate targets. The target was set by the government in Health Japan 21 (the second term) in 2012, based on the premise that “only smokers who want to quit smoking now, actually quit” [4].

**Fig. 1:**
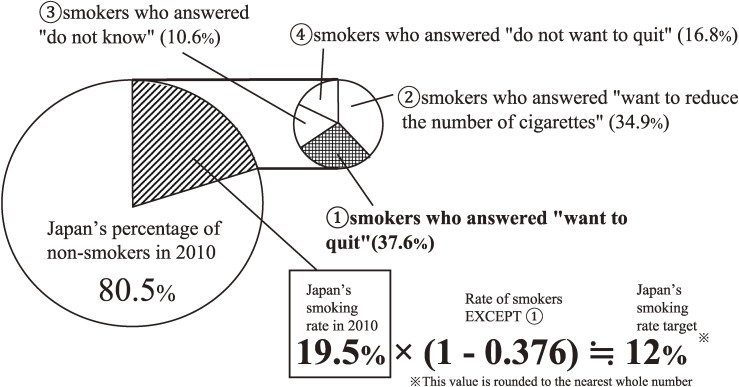
Premise of deciding Japanese smoking rate target This illustrates how the target smoking rate in Japan was established. The large pie chart represents the smoking rate in Japan in 2010 being 19.5%. The smaller pie chart indicates that 37.6% of smokers in the 2010 Japanese National Health and Nutrition Survey responded as “smokers who answered ‘want to quit’”. The multiplication formula demonstrates that the target smoking rate in Japan is calculated by subtracting the percentage of “smokers who answered ‘want to quit’” of all smokers in Japan from the 2010 smoking rate and multiplying the result.

In detail, using data from the 2010 Japanese National Health and Nutrition Survey, current adult smokers (prevalence = 19.5% in 2010) were categorized into four groups, identified by asking participants to choose one option from: “① I want to quit now” (37.6%), “② I want to reduce daily tobacco consumption” (34.9%), “③ I don’t know (10.6%)”, and “④ I don’t want to quit” (16.8%) [5]. If just the smokers who answered “① I want to quit now” (which was Japan’s definition of intention to quit) could quit smoking, Japan’s smoking rate target (12%) would be achieved (Fig. 1) [4]. This implies that the Japanese Ministry of Health, Labour and Welfare’s tobacco control policy focuses exclusively on smokers who answered “① I want to quit now” (37.6%). This figure is considerably lower than the 68% of smokers observed in an American survey who wanted to quit smoking [6, 7]. The discrepancy suggests that the definition of intention to quit varies between countries, and Japan’s smoking rate target is based on a narrower definition, focusing solely on smokers who actively express a current intention to quit.

However, it might be misleading to dismiss smokers who have expressed a desire to reduce their daily tobacco consumption or who are unsure about their quitting intentions as having no intention to quit. These categories may suggest a degree of dissatisfaction with their smoking habits making it inappropriate to assume that smokers who chose ② I want to reduce daily tobacco consumption and ③ I don’t know do not wish to quit smoking [8].

In Japan, heated tobacco products (HTPs) have become increasingly popular in recent years. Current smokers can be categorized into three tobacco use categories: exclusive combustible cigarette users, exclusive heated tobacco product (HTP) users, and dual (combustible cigarette and HTP) users [9, 10]. A previous study has shown that HTP users are less likely to want to quit smoking [11]. Despite the importance of considering the use of HTPs when studying smokers who want to quit, there is limited research that focuses on this aspect. Therefore, understanding the intention to quit by each tobacco users, including HTP users, is essential.

Hence, we aimed to re-define the category of smokers who intend to quit by including those with past intention to quit and estimate the proportion of potential quitters among total smokers. We also examined determinants of intention to quit, including daily tobacco consumption and tobacco use categories.

## Methods

### Study design and data collection

This cross-sectional internet-based study used data from The Japan “Society and New Tobacco” Internet Survey (JASTIS) [12]. A self-report questionnaire was commissioned by Rakuten Insight, a research company which maintains a pool of 2.6 million panelists and conducted between February 8–25, 2021 [13]. The study was reviewed and approved by the Research Ethics Committee of the Osaka International Cancer Institute (no. 1412175183) and the National Institute of Public Health (NIPH-IBRA#12112). The questionnaire was designed to collect demographic information of participants (e.g., sex, age, marital status, income) and, for those who smoked, specific data related to smoking habits (e.g., the tobacco use categories, attempts to quit smoking, frequency of smoking).

### Participants and selection criteria

**Fig. 2:**
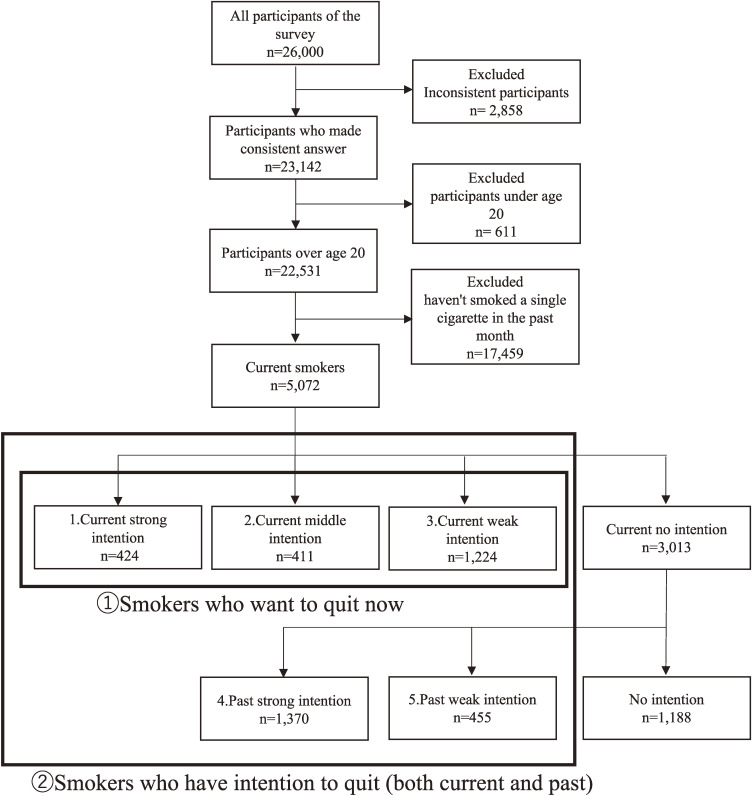
Data flow of selecting current smokers and definition of intention to quit This describes how from the respondents of the Japan “Society and New Tobacco” Internet Survey 2021, those not qualifying as Current smokers were excluded, and how Current smokers were defined. Additionally, Current smokers were further categorized into 1. Current strong intention, 2. Current middle intention, 3. Current weak intention, 4. Past strong intention, 5. Past weak intention, No intention. The sum of categories 1, 2, and 3 was defined as “① Smokers who want to quit now”. Then the sum of categories 1, 2, 3, 4, and 5 was defined as “② Smokers who have the intention to quit (both current and past)”.

A total of 26,000 participants from Japan aged 15–80 were initially included in the study. We excluded those who provided inconsistent answers (n = 2,858), those aged under 19 (n = 611), and selected current smokers (n = 5,072) from the remaining sample (Fig. 2). Current smokers were defined as individuals who smoked either combustible cigarettes or HTPs at least once a month. Inconsistent answers were identified based on three criteria: not choosing the second item from the bottom when instructed to do so, choosing every option in a list of 15 diseases, and choosing every item in a list of seven substances.

### Definition of current smokers who have intention to quit

We defined current smokers who have intention to quit as the total number of current smokers who want to quit now, current smokers who have attempted to quit and current smokers who have wanted to quit (on the basis that it is desirable not only for those who want to quit now but also for those who have wanted to quit in the past to succeed in the future).

Using responses from the JASTIS 2021 questionnaire (note: All questions must be answered so there is no missing data), their intention was divided into six categories based on Prochaska’s transtheoretical model [14]. Intention to quit was measured by asking participants, “Do you want to quit smoking now or not, and if so, when?” (note: This is question asked separately for combustible cigarettes and for HTPs). Past attempts were measured by the response to two questions; “Have you ever received smoking cessation treatment?” and “Have you ever tried to quit smoking?” and having wanted to quit in the past was measured by the response to “Have you ever considered quitting smoking?” (note: These questions do not differentiate between combustible cigarettes and HTPs).

Based on their responses, participants were categorized as follows:

First, we defined current smokers by their current quit intention:

1. Current strong intention: “I want to quit smoking within a month”.
2. Current middle intention: “I want to quit smoking within six months”
3. Current weak intention: “I want to quit but don’t think about when”.

The sum of categories 1, 2, and 3 was defined as “① Smokers who want to quit now” and the remaining categories were further divided into three, based on past quit attempts and intention to quit:

4. Past strong intention: “I tried to quit smoking” or “I have tried any type of smoking cessation treatment”
5. Past weak intention: “I have considered quitting smoking”

Then the sum of categories 1, 2, 3, 4, and 5 was defined as “② Smokers who have intention to quit (both current and past)”.

Current smokers who aren’t “② Smokers who have intention to quit (both current and past)” are defined as “No intention” (Fig. 2).

Note: smokers who used both combustible cigarettes and HTPs were considered dual users and were placed in the weaker intention category if they expressed different intentions for each product (e.g. combustible cigarettes: 2. Current middle intention and HTP: 4. Past strong intention→4. Past strong intention). The reason of this categorization is that it is important to clarify that we cannot conclude that quitting only one product would be sufficient for the health of these dual users. This is because both combustible cigarettes and HTPs are harmful [15].

### Variables

Tobacco-related variables included daily consumption of tobacco sticks (1–10, 11–20, over 20), other household smokers (0, ≥1), and tobacco use categories: exclusive combustible cigarette users, exclusive heated tobacco product (HTP) users, and dual (combustible cigarette and HTP) users. Additionally, based on previous studies [16, 17], we included socio-demographic factors such as sex (male, female), age (20–29, 30–39, 40–49, 50–59, 60–69, over 69 years), household income (first quartile, second quartile, third quartile, fourth quartile, prefer not to answer or do not know), marital status (married, never married or widowed/divorced), and self-rated health (poor, middle, good) as dependent variables. Daily consumption of HTP sticks was calculated by aggregating the number of each brand smoked. Note: Tobacco sticks include combustible cigarettes and HTPs. For dual users, daily consumption of tobacco sticks was calculated by summing the number of combustible cigarettes and HTPs.

### Statistical analysis

To investigate the relationship between intention to quit smoking and daily consumption of tobacco sticks and tobacco use categories, smokers were divided and compared in two ways: ① Smokers who want to quit now vs. others, ② Smokers who have intention to quit (both current and past) vs. others. Log-Poisson regression models were used to calculate adjusted odds ratios (aORs) and 95% confidence intervals for all variables, using R (version 4.0.5). In addition, as the multivariable models had convergence problems, we used a marginal structural binomial model to estimate adjusted ORs [18]. Statistical significance was set at p < 0.05. The percentages of ① Smokers who want to quit now and ② Smokers who have intention to quit were calculated. We then calculated the expected smoking rate in Japan if all ② Smokers who have intention to quit stop smoking in the future.

## Results

Table 1 describes the characteristics of the 5,072 current smokers who participated in this study. Among them, 424 (8.4% of current smokers) had 1. Current strong intention, 411 (8.1% of current smokers) had 2. Current middle intention, 1,224 (24.1% of current smokers) had 3. Current weak intention, 1,370 (27.0% of current smokers) had 4. Past strong intention, 455 (9.0% of current smokers) had 5. Past weak intention, and 1,188 (23.4% of current smokers) had No intention. 2,059 (40.6% of current smokers) were ① Smokers who want to quit now and 3,884 (76.6% of current smokers) were ② Smokers who have intention to quit (both current and past). If ② Smokers who have intention to quit (both current and past) stopped smoking in the future, the smoking rate would be 3.9%.

**Table 1:** Basic characteristics of study participants (current smokers)

**Characteristics**	**Total ** **smokers**	**1. Current ** **strong intention**	**2. Current ** **middle intention**	**3. Current ** **weak intention**	**4. Past ** **strong intention**	**5. Past ** **weak intention**	**6. No intention**	**P for difference^a^**
	**No.(% b)**	**No.(% b)**	**No.(% b)**	**No.(% b)**	**No.(% b)**	**No.(% b)**	**No.(% b)**	
Total smokers	5072 (100)	424 (8.4)	411 (8.1)	1224 (24.1)	1370 (27.0)	455 (9.0)	1188 (23.4)	

Tobacco use categories								<0.0001
Exclusive combustible cigarette users	2790 (55.0)	229 (54.0)	231 (56.2)	703 (57.4)	596 (43.5)	293 (64.4)	738 (62.0)	
Exclusive HTP users	932 (18.4)	82 (19.3)	85 (20.7)	249 (20.3)	277 (20.2)	62 (13.6)	177 (14.9)	
Dual users	1350 (26.6)	113 (26.7)	95 (23.1)	272 (22.2)	497 (36.3)	100 (22.0)	273 (23.0)	

Daily consumption of Tobacco sticks								<0.0001
0–10	2616 (51.6)	289 (68.2)	232 (56.4)	602 (49.2)	631 (46.1)	270 (59.3)	592 (49.8)	
11–20	1752 (34.5)	103 (24.3)	136 (33.0)	471 (38.5)	493 (36.0)	129 (28.4)	420 (35.4)	
over 20	704 (13.9)	32 (7.5)	43 (10.5)	151 (12.3)	246 (18.0)	56 (12.3)	176 (14.8)	

Sex								0.007763
Male	3646 (71.9)	303 (71.5)	272 (66.2)	874 (71.4)	998 (72.8)	310 (68.1)	889 (74.8)	
Female	1426 (28.1)	121 (28.5)	139 (33.8)	350 (28.6)	372 (27.2)	145 (31.9)	299 (25.2)	

Age group (years)								<0.0001
20–29	480 (9.5)	84 (19.8)	46 (11.2)	62 (5.1)	125 (9.1)	48 (10.5)	115 (9.7)	
30–39	697 (13.7)	65 (15.3)	60 (14.6)	136 (11.1)	205 (15.0)	71 (15.6)	160 (13.5)	
40–49	1280 (25.2)	110 (25.9)	92 (22.4)	291 (23.8)	367 (26.8)	116 (25.5)	304 (25.6)	
50–59	1187 (23.4)	85 (20.0)	95 (23.1)	309 (25.2)	307 (22.4)	93 (20.4)	298 (25.1)	
60–69	925 (18.2)	56 (13.2)	75 (18.2)	293 (23.9)	231 (16.9)	61 (13.4)	209 (17.6)	
Over 69	503 (9.9)	24 (5.7)	43 (10.5)	133 (10.9)	135 (9.9)	66 (14.5)	102 (8.6)	

Equivalent household income								0.08978
First quartile	1358 (26.8)	118 (27.8)	98 (23.8)	332 (27.1)	367 (26.8)	124 (27.2)	319 (26.9)	
Second quartile	1047 (20.6)	86 (20.3)	92 (22.4)	249 (20.3)	300 (21.9)	98 (21.5)	222 (18.7)	
Third quartile	1003 (19.8)	92 (21.7)	84 (20.4)	230 (18.8)	286 (20.9)	85 (18.7)	226 (19.0)	
Fourth quartile	904 (17.8)	66 (15.6)	61 (14.8)	208 (17.0)	250 (18.2)	82 (18.0)	237 (19.9)	
Prefer not to answer/do not know	760 (15.0)	62 (14.6)	76 (18.5)	205 (16.7)	167 (12.2)	66 (14.5)	184 (15.5)	

Self-rated health								<0.0001
Good	2869 (56.6)	197 (46.5)	206 (50.1)	683 (55.8)	765 (55.8)	293 (64.4)	725 (61.0)	
Middle	1507 (29.7)	131 (30.9)	141 (34.3)	373 (30.5)	390 (28.5)	116 (25.5)	356 (30.0)	
Poor	696 (13.7)	96 (22.6)	64 (15.6)	168 (13.7)	215 (15.7)	46 (10.1)	107 (9.0)	

Marital status								<0.0001
Married	3172(62.5)	258 (60.8)	271 (65.9)	778 (63.6)	900 (65.7)	282 (62.0)	683 (57.5)	
Never Married	1348(26.6)	134 (31.6)	101 (24.6)	297 (24.3)	305 (22.3)	122 (26.8)	389 (32.7)	
Widowed/divorced	552(10.9)	32 (7.5)	39 (9.5)	149 (12.2)	165 (12.0)	51 (11.2)	116 (9.8)	

Other household smokers								0.02747
0	3765(74.2)	312 (73.6)	288 (70.1)	899 (73.4)	999 (72.9)	352 (77.4)	915 (77.2)	
≥1	1307(25.8)	112 (26.4)	123 (29.9)	325 (26.6)	371 (27.1)	103 (22.6)	273 (22.8)	

^a^P for difference is calculated by Chi-square tests.

^b^Rounded to the second decimal place

Table 2 shows adjusted odds ratios (aORs) from the Log-Poisson regression analysis, which examines the association between intention to quit smoking and daily consumption of tobacco sticks, adjusting for covariates. Smokers who consume over 20 tobacco sticks daily are less likely to want to quit now than smokers of 1–10 tobacco sticks daily (aOR = 0.79, 95%CI = 0.71–0.87) and less likely to have intention to quit (both current and past) than smokers of 1–10 tobacco sticks daily (aOR = 0.93, 95%CI = 0.88–0.98). Smokers of 11–20 tobacco sticks daily are less likely to want to quit now than smokers of 1–10 tobacco sticks daily (aOR = 0.87, 95%CI = 0.82–0.93). Exclusive HTP users and dual users were less likely to want to quit now than exclusive combustible cigarette users (aOR = 0.88, 95%CI = 0.81–0.96) and (aOR = 0.84, 95%CI = 0.77–0.91). Exclusive HTP users and dual users were more likely to have intention to quit (both current and past) than exclusive combustible cigarette users (aOR = 1.09, 95%CI = 1.04–1.14) and (aOR = 1.07, 95%CI = 1.03–1.12).

**Table 2:** Log-Poisson Regression Analysis Results for Daily Tobacco Consumption: Estimating Odds Ratios

**Characteristics**	**Total smokers**	**① smokers who want to quit now**			**② smokers who have intention to quit ** **(both current and past)**		
	**No.(%)**	**No.(%)**	**Adjusted ORs(95%Cl)^b^**	**P for difference^a^**	**No.(%)**	**Adjusted ORs(95%Cl)^b^**	**P for difference^a^**
Total smokers	5072	2059 (40.6)	NA		3884 (76.6)	NA	

Tobacco use categories				<0.0001			<0.0001
Exclusive combustible cigarette users	2790 (55.0)	1163 (56.5)	1.0(reference)		2052 (52.8)	1.0(reference)	
Exclusive HTP users	932 (18.4)	416 (20.2)	**0.88(0.81–0.96)**		755 (19.4)	**1.09(1.04–1.14)**	
Dual users	1350 (26.6)	480 (23.3)	**0.84(0.77–0.91)**		1077 (27.7)	**1.07(1.03–1.12)**	

Daily consumption of Tobacco sticks				<0.0001			0.3348
1–10	2616 (51.6)	1123 (54.5)	1.0(reference)		2024 (52.1)	1.0(reference)	
11–20	1752 (34.5)	710 (34.5)	**0.87(0.82–0.93)**		1332 (34.3)	0.98(0.94–1.01)	
over 20	704 (13.9)	226 (11.0)	**0.79(0.71–0.87)**		528 (13.6)	**0.93(0.88–0.98)**	

Sex				0.05156			0.01093
Male	3646 (71.9)	1449 (70.4)	1.0(reference)		2757 (71.0)	1.0(reference)	
Female	1426 (28.1)	610 (29.6)	**1.13(1.05–1.21)**		1127 (29.0)	**1.06(1.02–1.11)**	

Age group(years)				0.006571			0.3875
20–29	480 (9.5)	192 (9.3)	1.0(reference)		365 (9.4)	1.0(reference)	
30–39	697 (13.7)	261 (12.7)	0.89(0.78–1.02)		537 (13.8)	0.99(0.92–1.07)	
40–49	1280 (25.2)	493 (23.9)	**0.81(0.71–0.91)**		976 (25.1)	0.97(0.91–1.04)	
50–59	1187 (23.4)	489 (23.7)	**0.83(0.73–0.94)**		889 (22.9)	0.95(0.88–1.02)	
60–69	925 (18.2)	424 (20.6)	0.93(0.81–1.06)		716 (18.4)	0.98(0.91–1.06)	
Over 69	503 (9.9)	200 (9.7)	0.90(0.77–1.05)		401 (10.3)	1.01(0.92–1.10)	

Equivalent household income				0.02409			0.1109
First quartile	1358 (26.8)	548 (26.6)	1.0(reference)		1039 (26.8)	1.0(reference)	
Second quartile	1047 (20.6)	427 (20.7)	1.07(0.98–1.18)		825 (21.2)	1.04(0.99–1.09)	
Third quartile	1003 (19.8)	406 (19.7)	1.07(0.97–1.18)		777 (20.0)	1.0(0.95–1.05)	
Fourth quartile	904 (17.8)	335 (16.3)	1.02(0.92–1.13)		667 (17.2)	**0.96(0.91–1.02)**	
Prefer not to answer/do not know	760 (15.0)	343 (16.7)	1.09(0.99–1.20)		576 (14.8)	1.01(0.96–1.07)	

Self-rated health				<0.0001			<0.0001
Good	2869 (56.6)	1086 (52.7)	1.0(reference)		2144 (55.2)	1.0(reference)	
Middle	1507 (29.7)	645(31.3)	1.07(0.997–1.15)		1151 (29.6)	**1.05(1.01–1.09)**	
Poor	696 (13.7)	328(15.9)	**1.24(1.13–1.35)**		589 (15.2)	**1.17(1.11–1.23)**	

Marital status				0.5152			<0.0001
Married	3172 (62.5)	1307 (63.5)	1.0(reference)		2489 (64.1)	1.0(reference)	
Never Married	1348 (26.6)	532(25.8)	**0.88(0.81–0.95)**		959 (24.7)	**0.89(0.85–0.93)**	
Widowed/divorced	552 (10.9)	220(10.7)	**0.89(0.80–0.98)**		436 (11.2)	0.97(0.92–1.03)	

Other household smokers				0.05869			0.01336
0	3765 (74.2)	1499 (72.8)	1.0(reference)		2850 (73.4)	1.0(reference)	
≥1	1307 (25.8)	560 (27.2)	0.97(0.90–1.04)		1034 (26.6)	1.01(0.97–1.05)	

Bold = statistical significance of p < 0.05.

a. P for difference is calculated by Chi-square tests.

b. Adjusted for listed all variables.

## Discussion

This is the first study to re-define the characteristics of current smokers who intend to quit and estimate their number. It outlines the relationship between daily tobacco consumption and the intention to quit while considering different tobacco use categories such as combustible cigarettes, HTPs, or both.

There is a gap between Japan’s smoking target rate (smoking prevalence = 12% by 2022) and that of other countries that have implemented tobacco control policies. For instance, Canada, Ireland and Australia are aiming to achieve a smoking prevalence of 5%, which is a common benchmark figure for tobacco control policies by 2035, 2025 and 2030 respectively [19–21]. While there are questions related to Australia’s likelihood of achieving its smoking target [22], such disparities raise the suggestion that Japan’s figure for the current smoking target rate may not be low enough. We found that heavy smokers, who consume more substantial quantities of tobacco per day, are less likely to want to quit now than lighter smokers. This data implies that the current approach of Japanese government might prioritize lighter smokers for quitting efforts, which can be justified to some extent. Categorizing smokers based on their intention to quit can be an effective means of providing targeted assistance for tobacco cessation, which aligns with the ‘Offer help to quit tobacco use’ principle of MPOWER, an anti-smoking strategy introduced by the WHO [23, 24].

Given these findings, we examined a broader definition of “wanting to quit” and suggest that Japan’s smoking target rate should be based on the premise that “only smokers who have intention to quit (both current and past) smoking, actually quit”. Our study reveals that heavy smokers are less likely to have intention to quit (both current and past) compared to lighter smokers. If Japan’s smoking target rate were based on the premise “only smokers who have intention to quit (both current and past) smoking (76.6%), actually quit”, the target smoking rate could be potentially reduced to 3.9% (based on the same premise as Fig. 1, considering smoking prevalence = 16.7% in 2019) [3], which is considerably below the current target of 12%. This calculation is done without changing the essential premise. Our proposed revision of the smoking target rate could serve as an invaluable reference for setting future targets in Japan. Our suggested target rate of 3.9% is guided by our study findings and closely matches the smoking prevalence targets (smoking prevalence = around 5%) adopted by other countries.

Although, as noted above, categorizing smokers based on their intention to quit can be effective for targeting assistance for quitting, this categorization should not serve as the basis for setting smoking rate targets. Our study found that heavy smokers are less likely to want to quit or have intention to quit (both current and past) than lighter smokers. This has critical implications for how Japan’s smoking prevalence targets are set: if these targets are based primarily on intention to quit among smokers, they may inadvertently favor lighter smokers who are more likely to possess this intention. Such an approach risks sidelining heavy smokers, who are at an increased risk for cardiovascular diseases and stroke due to their higher tobacco consumption [25].

Considering this, both the current premise of “only smokers who want to quit smoking now, actually quit” which is still suggested for implementation in Health Japan 21 (the third term) [26] and our suggested premise of “only smokers who have intention to quit (both current and past), actually quit” may not align with the objectives of Japan’s Health Promotion Act which considers the health status of the entire population [27]. The resultant target rate of 3.9% coincidentally aligns with the figure, less than 5%, set by many countries as the smoking rate target. It is constructed on the premise that “only smokers who have intention to quit (both current and past), actually quit” and not on the premise that “everyone should quit smoking”. We consider this premise as a second option, but we regard it as a pragmatic step towards lowering the smoking rate within the Japanese policy-making context.

Including smokers with a weak or no intention to quit, a comprehensive approach that reaches smokers across all societal strata, should be adopted. Various strategies are known to be effective, such as amplifying the effectiveness of warnings about the dangers of smoking [28], increasing tobacco taxes and prices [29, 30] and considering multi-channel approaches that include not only traditional mass media but also new social media platforms [31]. Canada, Ireland and Australia consider impacts on public health when determining smoking rate targets, irrespective of smokers’ intention to quit [21, 32, 33]. This comparison between the Japanese strategies and those of Canada, Ireland and Australia could serve as valuable references for other nations setting new smoking rate targets.

HTP users who have intention to quit (both current and past) face significant challenges in their efforts to quit smoking. Our study found that HTP users, both exclusive and dual users are more likely to have intention to quit (both current and past) smoking than exclusive combustible cigarette users. This is partly because many smokers who find it difficult to quit (despite having intention) believe that HTPs are less harmful than combustible cigarettes [34] and thus use them as a transitional stage in their cessation process, despite the fact that they contain certain substances in greater quantities that are more harmful than those in combustible cigarettes [35].

However, our study found that HTP users, both exclusive and dual users are less likely to want to quit smoking now than exclusive combustible cigarette users. This suggests that the intention to quit among HTP use might diminish over time, or the use of HTPs could be making the cessation process more challenging, aligned with previous research suggesting that the use of HTPs neither assists smokers to quit nor prevents former smokers from relapsing, indicating that HTPs should not be promoted as a cessation aid [36]. Combined with lenient regulation of HTPs in the media and in public places in Japan [37], these factors may impede the intention to quit among HTP users. This continues to be a significant public health challenge.

### Limitations

There are several limitations to this study. Firstly, the data was collected using an online survey, which may not be entirely representative of the Japanese population, although 82.9% of Japanese people have internet access [38]. Secondly, we relied on self-report questionnaires, which could be subject to recall and social desirability biases. In order to address these limitations, we excluded respondents with discrepancies from the analyses. Despite these limitations, this study offers valuable insights into the characteristics of Japanese smokers who have intention to quit.

## Conclusions

Our study is first to redefine the characteristics of smokers with intent to quit and estimate the proportion of total intention to quit, including HTP users, within the population. It challenges the current premise underpinning Japan’s smoking target rate. We examined a broader definition of ‘wanting to quit’, factoring in smokers with both current and past intention to quit; applying our definition could substantially reduce Japan’s smoking rate target from current 12.0% to 3.9%. We also highlight the struggles faced by HTP users attempting to quit, and advocate for stricter regulation and accurate media portrayals of HTP harm. These insights contribute to solving a significant public health challenge and should inform future smoking reduction policies.

## Abbreviations

HTP: Heated Tobacco Products
Dual users: Combustible cigarette and HTP users
JASTIS: Japan “Society and New Tobacco” Internet Survey
aOR: Adjusted Odds Ratio
Cl: Confidence Interval
NA: Not applicable
MPOWER: M: Monitor tobacco use and prevention policies and P: Protect people from tobacco smoke and O: Offer help to quit tobacco use and W: Warn about the dangers of tobacco and E: Enforce bans on tobacco advertising, promotion and sponsorship and R: Raise taxes on tobacco.

## Declarations

### Availability of data and material

The data can be accessed via the Japan “Society and New Tobacco” Internet Survey (JASTIS) study, Dr. Takahiro Tabuchi, upon reasonable request.
